# The efficacy of integrated hepatitis C virus treatment in relieving fatigue in people who inject drugs: a randomized controlled trial

**DOI:** 10.1186/s13011-023-00534-1

**Published:** 2023-04-24

**Authors:** Jørn Henrik Vold, Fatemeh Chalabianloo, Else-Marie Løberg, Christer F. Aas, Aaron G. Lim, Peter Vickerman, Kjell Arne Johansson, Lars Thore Fadnes

**Affiliations:** 1 Department of Addiction Medicine, Haukeland University Hospital, Jonas Lies Vei 65, N-5021 Bergen, Norway; 2 Department of Global Public Health and Primary Care, University of Bergen, Bergen, Norway; 3 Division of Psychiatry, Haukeland University Hospital, Bergen, Norway; 4 Department of Clinical Psychology, University of Bergen, Bergen, Norway; 5 Population Health Sciences, Bristol Medical School, University of Bristol, Bristol, UK

**Keywords:** Substance-related disorders, Fatigue, Opioid agonist therapy, Hepatitis C virus infection, Integrated treatment

## Abstract

**Background:**

Most people who inject drugs (PWIDs) suffer from severe fatigue, and chronic hepatitis C virus (HCV) infection may play a role in this. However, there is scarce evidence about interventions that alleviate fatigue among PWIDs. The present study investigated the effect of integrated HCV treatment on fatigue in this population compared to the effect of standard HCV treatment, adjusted for sustained virological response of the HCV treatment.

**Methods:**

This multi-center, randomized controlled trial evaluated fatigue as a secondary outcome of integrated HCV treatment (the INTRO-HCV trial). From May 2017 to June 2019, 276 participants in Bergen and Stavanger, Norway, were randomly assigned to receive integrated and standard HCV treatment. Integrated treatment was delivered in eight decentralized outpatient opioid agonist therapy clinics and two community care centers; standard treatment was delivered in specialized infectious disease outpatient clinics at referral hospitals. Fatigue was assessed prior to treatment and 12 weeks after treatment using the nine-item Fatigue Severity Scale (FSS-9). We applied a linear mixed model to evaluate the impact of integrated HCV treatment on changes in FSS-9 (ΔFSS-9) sum scores.

**Results:**

At baseline, the mean FSS-9 sum score was 46 (standard deviation (SD): 15) for participants on integrated HCV treatment and 41 (SD: 16) for those on standard treatment. Twelve weeks after completed HCV treatment, the mean FSS-9 sum score for participants receiving integrated HCV treatment was 42 (SD: 15) and 40 (SD: 14) for those receiving standard HCV treatment. Integrated HCV treatment did not reduce the FSS-9 scores compared to standard HCV treatment (ΔFSS-9: -3.0, 95% confidence interval (CI): -6.4;0.4).

**Conclusions:**

Fatigue is a common symptom among PWIDs. Integrated HCV treatment is at least equal to standard HCV treatment in improving fatigue.

**Trial registration:**

ClinicalTrials.gov.no NCT03155906, 16/05/2017.

**Supplementary Information:**

The online version contains supplementary material available at 10.1186/s13011-023-00534-1.

## Background

Fatigue is a debilitating symptom that affects as many as 50 to 80% of people with chronic hepatitis C virus (HCV) infection [1–3]. This contributes, in part, to extensive demotivation, non-restorative sleep, disinterest, lack of energy, and impaired quality of life, even among people in the early stages of HCV infection and those who achieve viral clearance [2, 4–6]. Among people who inject drugs (PWIDs), three out of four suffer from severe fatigue symptoms [7], which is comparable to those affected by stroke or major depressive disorder [8–10]. Multifactorial medical and psychosocial challenges––debt difficulties, extensive drug use, injecting drug use, mental disorders, and nutritional deficiency––dominate among these people and are associated with severe fatigue symptoms [7, 11, 12]. Additionally, HCV infection, which affects nearly half of PWIDs [13], is likely an essential cause of fatigue symptoms. Thus, investigating treatment approaches that might alleviate fatigue among PWIDs, particularly those infected with HCV is warranted.

In this regard, some studies have suggested that HCV treatment may reduce fatigue [14–17]. However, these mainly observational studies are encumbered substantially by a range of biases. Reaching PWIDs with HCV treatment may initiate other interventions concomitantly, such as addiction treatment and psychosocial support for debt, income, and housing stress, which are associated with changes in fatigue [7]. Thus, a randomized design is needed to disentangle the effect of HCV treatment from other confounding medical and psychosocial factors. Trials evaluating treatment models may be essential to explore the potential benefits of HCV treatment on fatigue among PWIDs. In a previous study from the INTRO-HCV trial, integrated HCV and addiction treatments involving decentralized outpatient clinics with multidisciplinary teams and close follow-up improved the sustained virological response (SVR) by 27% compared to standard HCV treatment for PWIDs [18]. Furthermore, the INTRO-HCV trial showed that the treatment initiation rate was 98% among participants who received integrated HCV treatment, compared to 77% among those who received standard HCV treatment. Based on the data from the same population [19], one could assume that fatigue in this group is particularly important for physical functioning and daily chores and commitments and could contribute to problems with these aspects. Thus, integrated HCV treatment may be preferable for reducing fatigue symptoms in this population.

This randomized controlled trial investigated the impact of integrated HCV infection treatment on fatigue using the nine-item fatigue severity scale (FSS-9) among PWIDs receiving oral direct-acting antivirals (DAAs) in western Norway. More specifically, we compared the impact of integrated HCV treatment to standard HCV treatment on changes of FSS-9 sum scores, adjusted for SVR.

## Methods

### Design and setting

The original study, the INTRO-HCV trial, was designed as a multi-center, randomized controlled trial [20]. This study evaluated fatigue as a secondary outcome of the INTRO-HCV trial. We recruited PWIDs with chronic HCV infection who were eligible for HCV treatment with DAAs in accordance with Norwegian HCV treatment guidelines (Additional file 1). Participants were recruited from eight outpatient clinics providing opioid agonist therapy (OAT) in Bergen and Stavanger, Norway, as well as two community care centers (CCCs) in Bergen providing primary healthcare to PWIDs. Enrollment was conducted from May 2017 to June 2019. For a more comprehensive description, a published protocol is available [20].

### Ethics approval and consent to participate

The present study was reviewed and approved by the Regional Ethical Committee for Health Research (REC) West, Norway (reference number: 2017/51/REK Vest, dated 29.03.2017/20.04.2017). All recruited participants were fully informed about the study, and their written informed consent was provided before their inclusion and randomization. All methods were carried out in accordance with relevant guidelines and regulations.

### Inclusion and exclusion criteria

Inclusion criteria were defined as follows: 1) receiving OAT opioids in the OAT outpatient clinics or people injecting drugs receiving healthcare from the two CCCs; 2) having chronic HCV infection defined as detecting HCV with HCV polymerase chain reaction in two separate blood samples drawn with an interval of at least six months; 3) eligibility for treatment according to the Norwegian HCV treatment guidelines; and 4) willingness to sign a written informed consent to participate in the trial. We excluded people who 1) currently received treatment for HCV; 2) were co-infected with human immunodeficiency virus (HIV) or hepatitis B virus (positive surface antigen) at the time of inclusion; 3) had severe extrahepatic manifestations (e.g., cryoglobulinemia or membranoproliferative glomerulonephritis); 4) had chronic renal disease stages 4–5 (glomerular filtration rate < 30 ml/min/1.73 m^2^); and 5) had decompensated liver disease (Child–Pugh class B or C). Additionally, people who did not complete the FSS-9 questionnaire during the study period were excluded. For details on demographic and clinical variables, see Table 1.

**Table 1:** Characteristics at baseline (n (%))

	**Integrated treatment**^**c**^ **(*n* = 141)**	**Standard treatment**^**c**^** (*n* = 135)**
*Age (years)*		
18–29	14 (10)	16 (12)
30–39	41 (29)	43 (32)
40–49	44 (31)	45 (33)
≥ 50	42 (30)	31 (23)
Median (IQR)	44 (36–52)	42 (34–49)
*Sex*		
Male	103 (73)	109 (81)
*Educational attainment*		
Not completed primary school	7 (5)	12 (9)
Completed primary school (9 years)	67 (48)	66 (49)
Completed high school (12 years)	52 (37)	44 (31)
Completed college or university	13 (9)	14 (10)
*Opioid agonist therapy*	120 (85)	120 (88)
*Unstable housing past 30 days*^a^	21 (14)	18 (13)
*Injected drug use past 12 months*	81 (58)	83 (64)
*Frequent drug use past 12 months*^b^		
Alcohol	35 (25)	32 (25)
Benzodiazepines	55 (40)	52 (41)
Cannabis	75 (54)	72 (56)
Opioids	17 (12)	15 (12)
Stimulants (amphetamines and cocaine)	48 (35)	39 (30)
*Infectious diseases*		
Hepatitis C virus genotypes		
1	47 (34)	44 (33)
2	< 10 (1)	< 10 (4)
3	91 (65)	80 (61)
4	< 5 (0)	< 5 (1)
6	< 5 (0)	< 5 (1)
Hepatitis B virus infection	0 (0)	0 (0)
Human immunodeficiency virus	0 (0)	< 5 (0)
*Liver stiffness*		
Transient elastography (≥ 12.5 kPa)	22 (16)	14 (11)
Aspartate transaminase to platelets ratio index (≥ 1.5)	13 (10)	13 (11)

The table displays the sociodemographic and clinical characteristics of participants randomly assigned to integrated and standard HCV treatment groups

*Legends*: *FSS-9* Nine-item fatigue severity scale, *IQR* Interquartile range, *kPa* Kilopascal

^a^Unstable housing was defined as living in a homeless shelter, with family or friends, or on the street during the 30 days leading up to the first health assessment (baseline)

^b^Frequent drug use was defined as using substance at least weekly during the 12 months leading up to the first health assessment (baseline)

^c^None basic characteristics were significantly different, comparing the integrated treatment group to the standard treatment group, with a significance level of 0.05

### Interventions

**Fig. 1:**
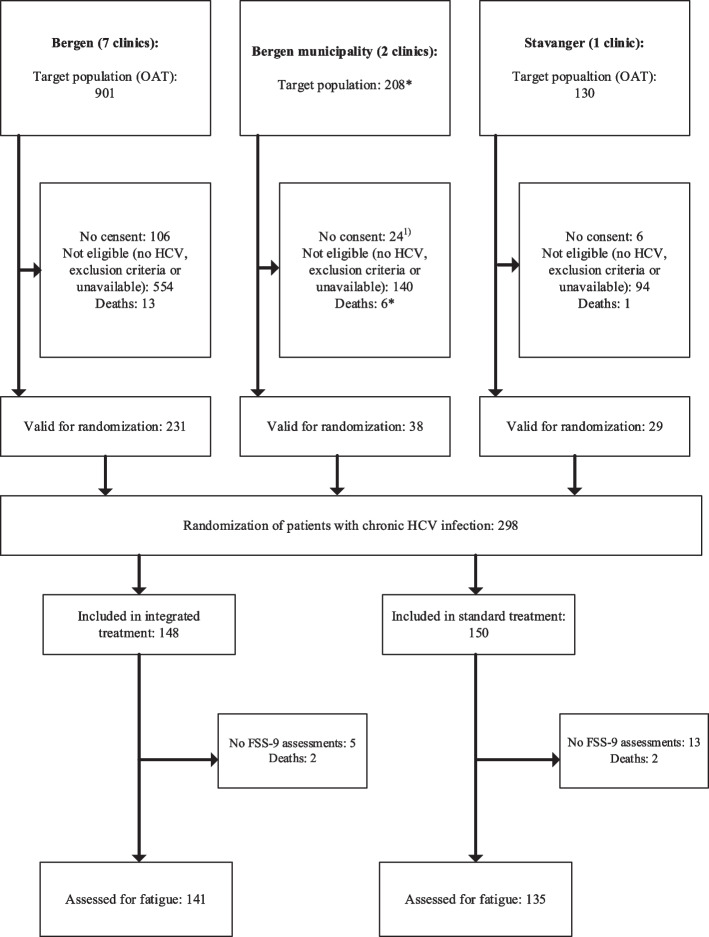
Trial profile for the study. Legends: ^1)^ Estimated numbers. FSS-9: Nine-item Fatigue Severity Scale; HCV: hepatitis C virus; OAT: opioid agonist therapy

A total of 148 participants were randomized into the integrated HCV treatment group and 150 into the standard HCV treatment group (Fig. 1). Ultimately, seven participants in the integrated treatment group and 15 in the standard treatment group were excluded due to death or lack of FSS-9 assessments. In total, 276 participants were included in the study – 141 in the integrated treatment group and 135 in the standard treatment group.

### Intervention – standard HCV treatment

Participants in the standard HCV treatment group were referred to the centralized outpatient infectious disease clinic at the collaborating referral hospital for HCV treatment. An appointment was given and usually scheduled within a few weeks after the referral; the participants were informed of this by mail. Their clinical assessment could involve additional blood samples and imaging before initiating HCV treatment. In the first year of the study, HCV consultation with a consultant in infectious diseases was mandatory, but with increasing clinical experience and growing evidence, the primary assessment became voluntary. Participants were offered follow-up assessments, including blood samples, during treatment in the infectious disease outpatient clinic every four weeks as well as a posttreatment assessment 12 weeks after completion. This typically involved a total of 4 to 5 consultation visits at the outpatient clinic. They were responsible for retrieving and adhering to their prescriptions, and attending assessment appointments. At 12 weeks after the end of treatment (EOT12), blood samples, including HCV polymerase chain reaction, were drawn at infectious disease outpatient clinics, OAT clinics, and CCCs. In addition, participants met at OAT clinics or CCCs to assess their FSS-9 levels.

In the standard HCV treatment, participants needed to travel to the hospital clinic and pay for the transport themselves, a distance that ranged from 1 to 25 km. They received standard follow-up in the OAT clinic for drug use disorders, and all other types of care – apart from HCV care – were integrated into the OAT follow-up. The OAT site staff encouraged participants to visit the infectious disease hospital clinics, but no further extensive follow-up was performed. There was a risk that scheduled appointments may overlap with other activities such as receiving OAT medication and other drug use treatment, since arrangements were not coordinated.

### Intervention – integrated HCV treatment

All assessments and medications for participants in the integrated treatment groups were provided onsite at the OAT clinics or CCCs, including DAAs, blood samples, and FSS-9 assessments. Compared with participants in the standard treatment group, participants in the integrated treatment group had no follow-ups in the referral hospital, and they received all assessments and medications at the local OAT clinics or CCC. In addition, they drew only two blood samples; prior to HCV treatment and at EOT12, and blood samples drawn every four weeks during the HCV treatment were not necessary. Integrated treatment was delivered at OAT clinics and CCC by multidisciplinary teams in both of the settings. The OAT clinics differed from the CCCs by offering OAT medications in addition to psychosocial approaches. The multidisciplinary teams at the OAT clinics were equipped with consultants in addiction medicine who were responsible for the OAT and other medical follow-ups and also psychologists providing mental health treatment. In both OAT and CCC settings, nurses and social workers, in cooperation with peer counselors, provided most of the participants’ daily follow-ups. All these professionals were existing clinical staff who closely worked together with the research nurses in management of the interventions and evaluations during the study period. For those eligible for HCV treatment, DAAs were administered by a nurse at OAT clinics/CCCs after a prescription from a consultant in infectious diseases. Contrary to standard HCV treatment, all HCV treatment and scheduled follow-ups during treatment were given in parallel with the observed intake of OAT medications and other care, in line with the study protocol. The number of deliveries of OAT and DAA medications per week was adapted to the level of functioning of each participant. For the most severely ill participants with the lowest level of daily functioning and high intake of multiple drugs, OAT medications and HCV treatment were usually dispensed daily in the OAT clinic, and intake was observed by a nurse. The multidisciplinary team planned assessments with participants, or drop-in approaches were applied.

### Data collection

Participants were evaluated prior to HCV treatment and EOT12 to record their health status, including fatigue level according to the FSS-9 score, sociodemographic data, current drug use, blood samples, transient elastography, and clinical examination. The health assessments were conducted by specialized research nurses in close collaboration with the clinics’ consultants in addiction medicine and infectious diseases. A medical team followed up with those who did not meet the criteria for inclusion in the study. Data from the health assessments prior to and after HCV treatment were defined as the study’s baseline and EOT12 (endpoint), respectively.

### Randomization and masking

Selected participants were randomized at a 1:1 ratio using blocks of 10 stratified by city and assigned into integrated (*n* = 148) or standard treatment (*n* = 150) for the trial. Complete blinding was considered impractical and would have reduced external validity [21], although some masking measures were taken [20]. In short, randomization was disclosed to clinical staff providing treatment and follow-up. Participants were informed of key elements in the delivery of the respective intervention and follow-up to which they were assigned, but no information was shared on treatment and follow-up alternatives or the hypotheses for the study.

### Measurement

We assessed fatigue using the FSS-9, including items considering mental and physical functioning, motivation, carrying out duties, and interfering with work, family, or social life. The FSS-9 is a well-known questionnaire to quantify fatigue during the week prior to the assessment [22–27], with high validity and reliability in people undergoing HCV treatment [28]. The FSS-9 items are answered on a Likert scale ranging from 1, no fatigue, to 7, worst fatigue, demonstrating the fatigue level. A high FSS-9 score indicates a high level of fatigue; a mean score greater than 4.0 reveals severe fatigue [27]. A nine-item fatigue severity scale sum score was calculated by summarizing the points generated by the nine items. The FSS-9 employed had been translated and back-translated from US-English into Norwegian by qualified native Norwegian-speaking translators (Additional file 2) [29].

We drew blood samples, including hepatitis B virus surface antigen, HIV antigen/antibodies, thrombocytes, and aspartate aminotransferase, as well as HCV antibodies and HCV polymerase chain reactions. Liver stiffness was measured by calculating the aspartate aminotransferase to platelet ratio index and performing transient elastography at baseline (Additional file 3). Transient elastography calculates liver stiffness using the median value of ten repeated measurements on an empty stomach [30, 31].

### Statistical analyses

We used Stata SE version 17 (StataCorp, TX, USA) for descriptive analyses and linear mixed model analyses, and IBM SPSS version 26.0 for expectation–maximization calculation. The threshold for statistical significance was set to *p* < 0.05 for all analyses unless otherwise stated. All statistical analyses were conducted following CONSORT and SPIRIT guidelines [32, 33]. The sample size was calculated for the primary outcome of SVR in the INTRO-HCV trial [20].

We dealt with any missing values in FSS-9 scores at baseline and EOT12 as “missing at random” when running expectation–maximization algorithm [34, 35]. We identified missing values in 1.4% of FSS-9 scores at baseline and 29.9% at EOT12, and all were replaced with estimated values. The expectation–maximization algorithm for computing data iteratively performs maximum likelihood estimation in the presence of latent variables [36], recommended for optimizing the mixed models. Sensitivity analyses without estimated values were conducted in all regression models.

The FSS-9 sum scores at baseline and EOT12 were calculated as described above (“Measurement” section). We created Pen’s parades in which the FSS-9 sum score at baseline were in sorted order from lowest to highest values and spikes were performed to express changes in FSS-9 scores from baseline to EOT12 in the integrated and standard HCV treatment groups. Additionally, linear mixed models were applied to investigate whether the predictor variables of treatment groups (dichotomized as standard (0) versus integrated (1)), and SVR (dichotomized as no (0) versus yes (1)), defined as undetectable HCV RNA 12 weeks after HCV treatment completion, affected the ΔFSS-9 sum scores from baseline to EOT12. The linear mixed models were random intercept fixed slope regression models. The restricted maximum likelihood was set as the estimator [37, 38]. The full information of maximum likelihood ensured that all available FSS-9 sum scores were used. The linear mixed model analysis was performed as intention-to-treat and per-protocol analyses and as a sensitivity analysis without computed data. In addition, linear mixed model sensitivity analyses were performed to evaluate whether achieving SVR affected the FSS-9 sum score, adjusted for sex, age, educational attainment, injecting drug use, and drug use.

## Results

### Characteristics at baseline

The median age was 44 years (interquartile range (IQR): 36–52) in the integrated HCV treatment group. Of those, 73% were male, and 58% had injected drugs recently. In the standard HCV treatment group, the median age was 42 years (IQR 34–49), 81% were male, and 64% had injected drugs recently. HCV genotype 3 was most prevalent, representing 65% of participants in the integrated HCV treatment group and 61% in the standard HCV treatment group.

### FSS-9 sum scores at baseline and EOT12

At baseline, the mean FSS-9 sum score for participants on receiving integrated treatment was 46 (Standard deviation (SD): 15) and 41 (SD: 16) for those on standard treatment. The mean FSS-9 sum score in both groups was slightly left-skewed and tended toward a flattened distribution at baseline (Additional file 4). At EOT12, the mean FSS-9 sum score for participants receiving integrated treatment was 42 (SD: 15) and 40 (SD: 14) for those receiving standard treatment. For detailed information on the FSS-9 sum scores at baseline and EOT12, see Additional file 5.

### The impact of integrated HCV treatment on change in the FSS-9 sum score, adjusted for SVR

**Fig. 2:**
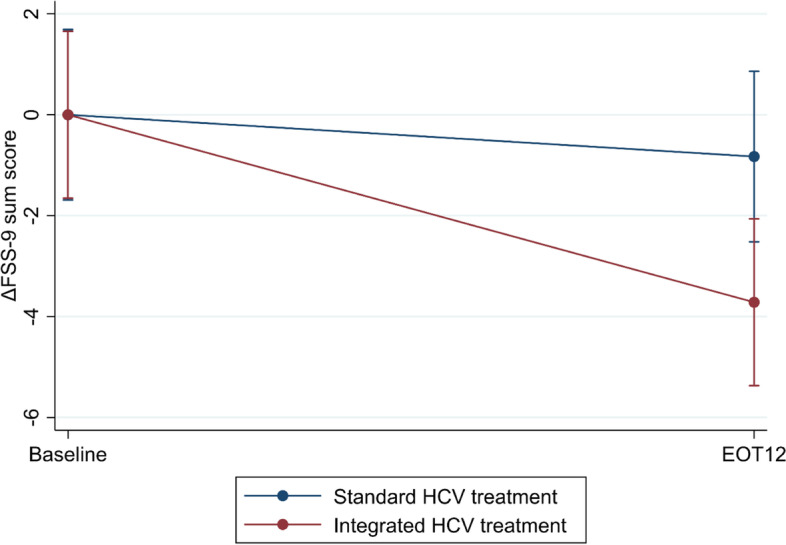
A linear prediction of changes in FSS-9 sum scores from baseline to EOT12 (intention-to-treat analysis) (*N* = 276). Legends: The figure displays the linear prediction (fixed portion) including 95% confidence intervals of FSS-9 sum score (ΔFSS-9 sum score) at baseline and from baseline (prior to HCV treatment) to EOT12 for integrated and standard HCV treatment groups. The FSS-9 sum score ranges from 9 points, no fatigue, to 63 points, worst fatigue. EOT12: 12 weeks after the end of HCV treatment; FSS-9: Nine-item fatigue severity scale; HCV: Hepatitis C virus

**Fig. 3:**
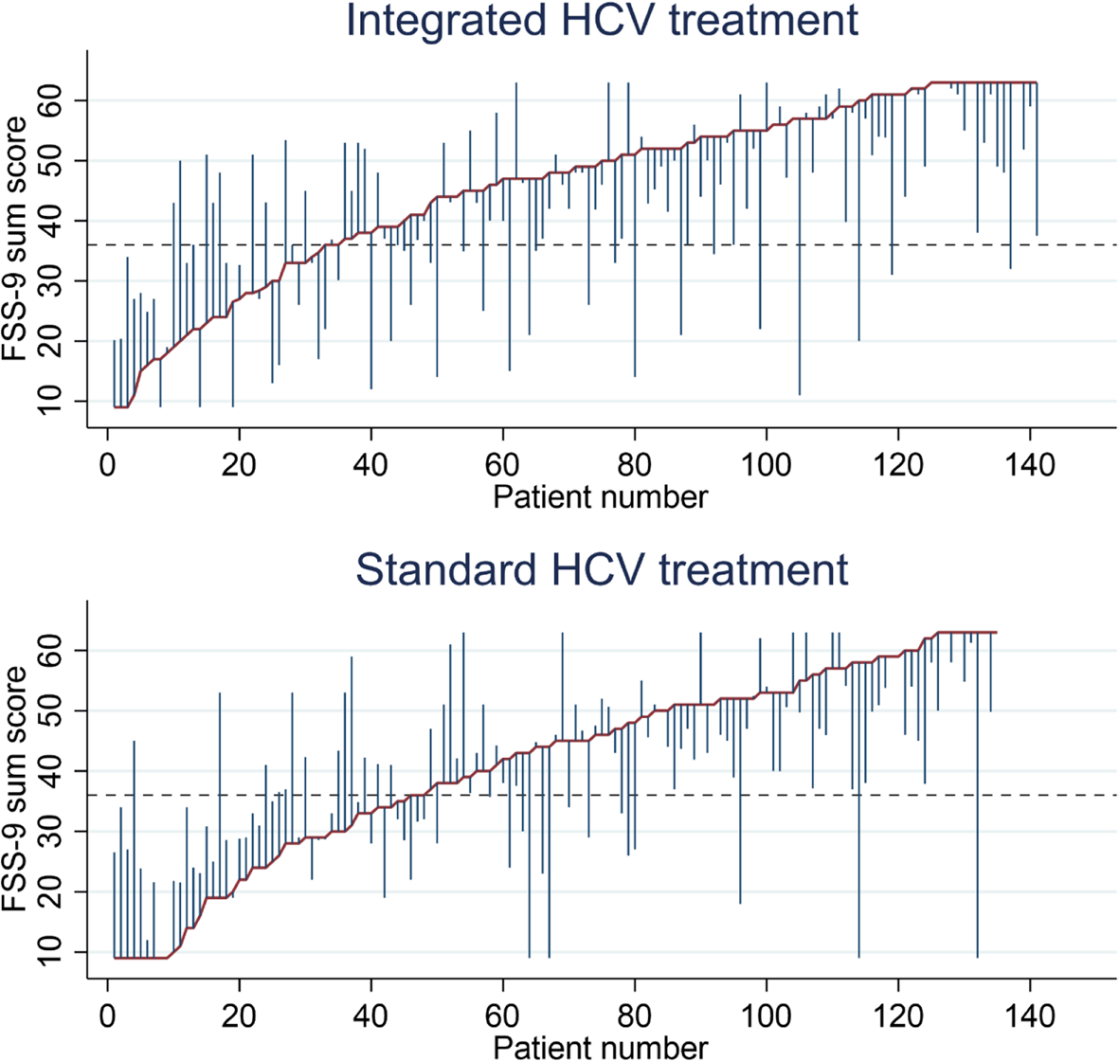
Pen’s parades of FSS-9 sum scores at baseline and EOT12 (*N* = 276). Legends: The figures display participants who received integrated and standard HCV treatment. The graphs demonstrate the FSS-9 sum scores at baseline/prior to the HCV treatment and EOT12. The red line represents the FSS-9 sum scores at baseline when the participants are in sorted order by FSS-9 sum scores (from lowest (left) to highest (right) score). The blue spikes demonstrate the FSS-9 sum score at EOT12. The length of the spikes mark the changes in the FSS-9 sum score from baseline to EOT12. Participants without spikes did not complete FSS-9 assessment at EOT12. The dotted line demonstrates the cut-off value for severe fatigue (36 points). EOT12: 12 weeks after the end of treatment; FSS-9: Nine-item Fatigue Severity Scale. EOT12: 12 weeks after the end of treatment; FSS-9: Nine-item Fatigue Severity Scale

Integrated HCV treatment did not reduce the FSS-9 sum score from baseline to EOT12 more than standard HCV treatment (ΔFSS-9 sum score: –3.0, 95% confidence interval (CI): –6.4; 0.4) (Table 2, Fig. 2) (intention to treat). Moreover, substantial intraindividual variations in FSS-9 sum scores over time were observed in both groups (Fig. 3). Likewise, per-protocol and sensitivity analyses without computed data showed similar results (Additional files 6, 7, 8, 9 and 10). Achieving SVR was not associated with changes in the FSS-9 sum score from baseline to EOT12, adjusted for sociodemographic factors, injecting drug use, and types of drugs used (Additional files 11 and 12).

**Table 2:** Linear mixed model of ΔFSS-9 sum scores from baseline to EOT12 for integrated HCV treatment (intention-to-treat) (*N* = 276)

	**Effect estimates**	
	**Coefficient (95% CI)**	***p*-value**
Time trend	–1.2 (–4.2;1.8)	0.422
Δ*FSS-9 sum score from baseline to EOT12*		
Standard HCV treatment	0.0 (ref.)	-
Integrated HCV treatment	–3.0 (-6.4;0.4)	0.083
Achieving SVR	0.6 (-2.2;3.4)	0.665

The table displays a linear mixed model analysis (Restricted Maximum Likelihood) regression of the impact of integrated HCV treatment on changes in FSS-9 sum scores (ΔFSS-9 sum score) from baseline to EOT12 (intention-to-treat analysis), adjusted for achieving SVR at EOT12. The FSS-9 sum score ranges from 9 points, no fatigue, to 63 points, worst fatigue

*Legends*: *EOT12* 12 weeks after the end of HCV treatment, *FSS-9* Nine-item fatigue severity scale, *SVR* Sustained virological response

## Discussion

The present RCT demonstrated that, compared to standard HCV treatment, integrated HCV treatment did not reduce fatigue from baseline to EOT12 among PWIDs; however, a non-significant improvement in the fatigue level was observed. The fatigue level was high in both the integrated and the standard HCV treatment groups, with substantial intraindividual variation from baseline to EOT12.

To our knowledge, this was the first trial conducted in outpatient OAT clinics and CCCs to investigate the impact of integrated HCV treatment on fatigue among PWIDs. Although no significant improvement in integrated HCV treatment compared to standard treatment was found, we revealed non-significant reduction in FSS-9 scores with integrated HCV treatment. This implies that an integrated approach is at least equal to or possibly more effective than standard HCV treatment in reducing fatigue symptoms in this population. Achieving SVR representing 85% and 64% of participants in integrated and standard HCV treatments, respectively, according to the INTRO-HCV trial [18], not contributed to the improved fatigue level. In contrast, two cohort studies in which people co-infected with HIV and HCV recruited showed that DAA treatment may reduce fatigue symptoms [14, 15]. However, liver cirrhosis, representing up to 45% of these participants, and co-infection with HIV could have influenced the results of those studies [16, 17]. Liver cirrhosis caused by HCV infection is associated with fatigue [39], and mono-infection by HCV is associated with more fatigue than co-infection of HCV and HIV [17], arguably due to closer follow-ups of co-infected people. In our PWID population, few participants had liver cirrhosis, and no participants were co-infected with HIV, which could explain our results.

The present study demonstrated that integrated HCV treatment was at least equal to relieving fatigue symptoms among PWIDs than standard HCV treatment, adjusted for achieving SVR. The results align with existing literature on this topic [40, 41]. Although the impact of integrated HCV treatment on fatigue was not found to be superior to standard HCV treatment in the present study, the integrated HCV treatment approach improved medical treatment among PWIDs significantly, as demonstrated in the INTRO-HCV trial [18]. Due to many within this population experiencing challenging life situations, close follow-ups and decentralized treatment are essential to provide healthcare and improve their medical and psychosocial conditions [18]. In a cohort study in which fatigue was evaluated in people with drug use disorders, benzodiazepine, cocaine, or amphetamine use, debt difficulties, and female sex were significantly associated with fatigue [7]. Otherwise, people with a higher HCV viral load (≥ 800,000 IU/ml) had more fatigue than those with a lower HCV viral load (< 800,000 IU/ml) prior to HCV infection treatment; however, other studies did not find a similar association based on clinical and histological features [42–45]. Even though the studies are equivocal on the impact of underlying medical and psychosocial challenges on fatigue among PWIDs, as demonstrated in the present study and the INTRO-HCV trial [18], it is reasonable that integrated HCV treatment increases adherence to HCV treatment and may improve psychosocial challenges by multidisciplinary teams providing close follow-ups. Thus, integrated HCV treatment may be conceptually better suited to reach PWIDs with other interventions, such as adequate addiction treatment, which is associated with changes in fatigue levels [7, 46].

The integrated and standard treatment groups demonstrated substantial intraindividual variation in fatigue levels over time. This corresponds with the results detected in another fatigue study of people infected with HCV [28]. The large intraindividual variation in the present study is likely attributable to changes in housing- and debt stress, comorbid mental disorders, and drug overdoses and withdrawals that necessitate hospitalizations, which are significantly associated with fatigue [7]. Although the fatigue assessments were performed under medically and psychosocially stable conditions and randomized controlled trial design was used, it was hard to eliminate all the various influencing factors; and thus, some intraindividual variations in fatigue level were expected [18]. However, sensitivity analyses of our study sample of which sociodemographic factors, injecting drug use, and types of drugs used were considered showed no association between achieving SVR and changes in fatigue level, in line with the present study’s primary findings. This reflects the complexity of interpreting the impact of interventions on fatigue among PWIDs, even with targeted HCV treatment interventions.

## Strengths and limitations

A major strength of this study is its trial design of individual randomization with balanced groups, which minimizes potential confounding. Furthermore, we included PWIDs who usually struggle with adherence to standard HCV treatment and have frequently discontinued previous HCV assessment and treatment in centralized infectious disease outpatient clinics. A limitation of this study is in the selection of outpatient clinics, where most participants received OAT to recover from opioid dependence, affecting the generalizability of our results to non-OAT populations. Another limitation is the almost 30% loss-to-follow-up of the FSS-9 assessment at EOT12 and the exclusion of 18 randomized participants due to missing FSS-9 assessments during the period. This may explain the five-point higher FSS-9 sum score in the intervention group than in the control group at baseline. Furthermore, due to system and individual delays and changes in national guidelines for HCV treatment throughout the study period, the FSS-9 assessments were not conducted in exact concurrence with HCV treatment initiation and EOT12. This could affect the interpretation of the predicted fatigue changes from baseline. Furthermore, the FSS-9 did not consider specific issues related to completing the questionnaire, such as cognitive impairments and physical disabilities. These issues could introduce information and recall bias of reported fatigue symptoms. Moreover, a time-to-treatment analysis from the first fatigue measurement to the HCV treatment initiation could be performed to adjust for changes in fatigue. However, the fatigue level was assumed to be substantially unchanged during the few weeks from the first health assessment to the HCV treatment initiation.

## Conclusion

The present trial documented that fatigue is a common symptom among PWIDs. Integrated HCV treatment was at least equal to standard HCV treatment in alleviating fatigue. Integrated HCV treatment may be a treatment approach in other medical and psychosocial care to improve fatigue.

## Supplementary Information

**Additional file 1.** Norwegian national HCV treatment guidelines during the study period. 

**Additional file 2.** The US-English and the Norwegian versions of the FSS-9. Legends: FSS-9; Nine-items Fatigue Severity Scale. All items in the FSS-9 are ranged as a Likert scale from 1 to 7, where 1 indicates “strongly disagree” and 7 indicates “strongly agree”. 

**Additional file 3.** Aspartate aminotransferase to platelet ratio index. Legends: The figure displays the equation to calculate APRI score. AST upper limit of normal range was defined as 45 IU/Land 35 IU/L. 

**Additional file 4.** Distribution of FSS-9 sum scores for integrated HCV treatmentand standard HCV treatmentgroups at baseline. Legends: The two graphsdisplay the FSS-9 sum scores for integrated HCV treatmentand standard HCV treatmentat baseline. The red lines demonstrate the distribution of the FSS-9 sum scores with skewness –0.8and –0.5and kurtoses 2.7 and 2.3. The FSS-9 sum scores ranges from 9 points, no fatigue, to 63 points, worst fatigue. 

**Additional file 5.** Primary end point analyses of mean and sum scores of FSS-9 at baseline and EOT12 Legends: The table displays the mean and sum scores of FSS-9 at baseline and EOT12 among participants who were included in the intention-to-treat and per-protocol analyses, respectively. The FSS-9 sum score ranges from 9 points, no fatigue, to 63 points, worst fatigue. Each item was ranged on a Likert scale from 1 point, no fatigue, to 7 points, worst fatigue. EOT12: 12 weeks after the end of treatment; SD: Standard deviation. 

**Additional file 6.** Linear mixed model of ΔFSS-9 sum score from baseline to EOT12 for integrated HCV treatment (per-protocol) (number of participants = 212, number of observations: 424). Legends: The table displays a linear mixed model analysis (Restricted Maximum Likelihood) regression of the impact of integrated HCV treatment on changes in FSS-9 sum scores (ΔFSS-9 sum scores) from baseline to EOT12 (per-protocol analysis), adjusted for acheiving SVR at EOT12. The FSS-9 sum score ranges from 9 points, no fatigue, to 63 points, worst fatigue. EOT12: 12 weeks after the end of HCV treatment; FSS-9: Nine-item fatigue severity scale.

**Additional file 7.** A linear prediction of changes in FSS-9 sum scores from baseline to EOT12. Legends: The figure displays the linear predictionincluding 95 % confidence intervals of changes in FSS-9 sum scorefrom baseline to EOT12 for integrated and standard HCV treatment groups. EOT12: 12 weeks after the end of HCV treatment; FSS-9: Nine-item fatigue severity scale; HCV: Hepatitis C virus. 

**Additional file 8.** Pen’s parades of FSS-9 sum scores at baseline and EOT12. Legends: The figures display participants who received integrated and standard HCV treatment and were included in the per-protocol analysis. The graphs demonstrate the FSS-9 sum scores at baseline/prior to the HCV treatment and EOT12. The red line represents the FSS-9 sum scores at baseline when the participants are in sorted order by FSS-9 sum scores to highestscore). The blue spikes demonstrate the FSS-9 sum score at EOT12. The length of the spikes mark the changes in the FSS-9 sum score from baseline to EOT12. Participants without spikes did not complete FSS-9 assessment at EOT12. The dotted line demonstrates the cut-off value for severe fatigue. EOT12: 12 weeks after the end of treatment; FSS-9: Nine-item Fatigue Severity Scale.  

**Additional file 9.** Linear mixed model of ΔFSS-9 sum scores from baseline to EOT12 for integrated HCV treatment (intention-totreat, sensitivity analysis without computed data), adjusted for SVR at EOT12 (number of participants = 189, number of observations: 378). Legends: The table displays a linear mixed model analysis (Restricted Maximum Likelihood) regression of the impact of integrated HCV treatment and SVR on changes in FSS-9 sum scores (ΔFSS-9 sum score) from baseline to EOT12 (intention-to-treat analysis without computed data by the expectation–maximization algorithm). The FSS-9 sum score ranges from 9 points, no fatigue, to 63 points, worst fatigue. EOT12: 12 weeks after the end of HCV treatment; FSS-9: Nine-item fatigue severity scale; SVR: Sustained virological response.   

**Additional file 10.** Linear mixed model of ΔFSS-9 sum scores from baseline to EOT12 for integrated HCV treatment, adjusted for SVR . Legends: The table displays a linear mixed model analysis regression of the impact of integrated HCV treatment and SVR on changes in FSS-9 sum scoresfrom baseline to EOT12. The FSS-9 sum score ranges from 9 points, no fatigue, to 63 points, worst fatigue. EOT12: 12 weeks after the end of HCV treatment; FSS-9: Nine-item fatigue severity scale; SVR: Sustained virological response.  

**Additional file 11.** Linear mixed model of the association between sociodemographic factors, injecting drug use, and drug use and FSS-9 sum score. Legends: The table displays a linear mixed model analysis regression of the impact of sociodemographic factors, injecting drug use, and drug use on FSS-9 sum scores at baseline and from baseline to EOT12in the study sample. The FSS-9 sum score ranges from 9 points, no fatigue, to 63 points, worst fatigue. “Educational attainment” was defined as the highest level of education completed. “Injecting substance use” was defined as having injected any substance at least once during the 12 months leading up to the first health assessment. Drug use was categorized according to the use during the past year. Frequent drug use was defined as consuming at least one of the drugs in the five drug classes more than weekly during the year leading up to the first health assessment. Participants who did not use drugs or used them less than weekly during the year were categorized as having “no frequent use of drugs”. Missing values were identified in 1.4% of FSS-9 scores, 1.1 % of educational attainment, 2.5 % of injecting drug use and 3.3 % of drug use at baseline and 29.9% of FSS-9 score at EOT12, and all were handled as “missing at random” and replaced with estimated values using expectation- maximization algorithm. Except for the “achieving SVR” predictor, we kept all the predictor variables constant at the baseline level in predicting changes in the FSS-9 sum scores from baseline to EOT12. To explore whether predictors predicted changes in the FSS-9 score from baseline to EOT12, the interaction between these factors and timeand EOT12) were added. EOT12: 12 weeks after the end of HCV treatment; FSS-9: Nine-item fatigue severity scale; HCV: Hepatitis C virus; SVR: Sustained virological response. 

**Additional file 12.** Linear mixed model of the association between sociodemographic factors, injecting drug use, and drug use and FSS-9 sum score. Legends: The table displays a linear mixed model analysis regression of the impact of sociodemographic factors, injecting drug use, and drug use on FSS-9 sum scores at baseline and from baseline to EOT12in the study sample. The FSS-9 sum score ranges from 9 points, no fatigue, to 63 points, worst fatigue. “Educational attainment” was defined as the highest level of education completed. “Injecting substance use” was defined as having injected any substance at least once during the 12 months leading up to the first health assessment. Drug use was categorized according to the use during the past year. Frequent drug use was defined as consuming at least one of the drugs in the five drug classes more than weekly during the year leading up to the first health assessment. Participants who did not use drugs or used them less than weekly during the year were categorized as having “no frequent use of drugs”. Missing values were identified in 1.4% of FSS-9 scores, 1.1 % of educational attainment, 2.5 % of injecting drug use and 3.3 % of drug use at baseline and 29.9% of FSS-9 score at EOT12, and all were handled as “missing at random” and replaced with estimated values using expectation- maximization algorithm. Except for the “achieving SVR” predictor, we kept all the predictor variables constant at the baseline level in predicting changes in the FSS-9 sum scores from baseline to EOT12. To explore whether predictors predicted changes in the FSS-9 score from baseline to EOT12, the interaction between these factors and timeand EOT12) were added. EOT12: 12 weeks after the end of HCV treatment; FSS-9: Nine-item fatigue severity scale; HCV: Hepatitis C virus; SVR: Sustained virological response. 

## Data Availability

The datasets analyzed during the current study are not publicly available due data protection requirements but are available from the corresponding author on reasonable request.

## Abbreviations

CCC: Community care centers
CI: Confidence interval
DAA: Direct-acting antiviral agent
EOT12: 12 Weeks after the end of treatment
FSS-9: Nine-item fatigue severity scale
HCV: Hepatitis C virus
HIV: Human immunodeficiency virus
IQR: Interquartile range
OAT: Opioid agonist therapy
PWID: People who inject drugs
SD: Standard deviation
SVR: Sustained virological response
