# Supplementary material for: The efficacy of integrated hepatitis C virus treatment in relieving fatigue in people who inject drugs: a randomized controlled trial
Source: Subst Abuse Treat Prev Policy. 2023 Apr 24;18:25. doi: 10.1186/s13011-023-00534-1 (PMC10123982; doi:10.1186/s13011-023-00534-1)
Supplement: Supplementary file 1 — Additional file 1. Norwegian national HCV treatment guidelines during the study period. [file 13011_2023_534_MOESM1_ESM.pdf]

Additional File 1

Several guideline documents on HCV treatment in Norway have been relevant in the period 2017–2019. The following sources are some of the key documents in choice of treatment (most are in Norwegian and some of these have restricted access):

Guidelines from the Norwegian association for infectious diseases, version 7 (2017):
https://www.hepatittfag.no/pdf-hcv

Version 8 (2019):
https://www.legeforeningen.no/contentassets/7bdf07c45b95411aa3c66e27822da583/veilederrevi
sjon-8-1.pdf

The treatment choice guidelines from the health authorities:
Period 01.03.2016–28.02.2017: http://innsiden.helse-
bergen.no/komiteer/legemiddelkomiteen/_layouts/WopiFrame.aspx?sourcedoc=/komiteer/legemi
ddelkomiteen/Dokumentbibliotek%20lager/Informasjon%20om%20LIS-anbud/2016/LIS-
anbefalinger%20Hepatitt%20C%202016%20-
%20HBe.docx&action=default&Source=http%3A%2F%2Finnsiden%2Ehelse%2Dbergen%2Eno
%2Fkomiteer%2Flegemiddelkomiteen%2FDokumentbibliotek%2520lager%2FForms%2FAllIte
ms%2Easpx%3FRootFolder%3D%252Fkomiteer%252Flegemiddelkomiteen%252FDokumentbi
bliotek%2520lager%252FInformasjon%2520om%2520LIS%252Danbud%252F2016%26Folder
CTID%3D0x0120008729410275231245591CBCF04418E246C%26View%3D%7B4E86623D%2
D3B87%2D484B%2DA087%2D07D7BB213411%7D&DefaultItemOpen=1

Period 01.03.2017–28.02.2018: http://innsiden.helse-
bergen.no/komiteer/legemiddelkomiteen/_layouts/WopiFrame.aspx?sourcedoc=/komiteer/legemi
ddelkomiteen/Dokumentbibliotek%20lager/Helseforetakenes%20LIS%20HCV%20anbefalinger
%202017%20-
%20reviderte%20130617.pdf&action=default&Source=http%3A%2F%2Finnsiden%2Ehelse%2
Dbergen%2Eno%2Fkomiteer%2Flegemiddelkomiteen%2FDokumentbibliotek%2520lager%2FF
orms%2FAllItems%2Easpx&DefaultItemOpen=1

Period 01.02.2018–31.01.2019: http://innsiden.helse-
bergen.no/komiteer/legemiddelkomiteen/_layouts/WopiFrame2.aspx?sourcedoc=/komiteer/legem
iddelkomiteen/Dokumentbibliotek%20lager/Informasjon%20om%20LIS-
anbud/2018/LIS%20HCV%20anbefalinger%202018.pdf&action=default&Source=http%3A%2F
%2Finnsiden%2Ehelse%2Dbergen%2Eno%2Fkomiteer%2Flegemiddelkomiteen%2FDokumentb
ibliotek%2520lager%2FForms%2FAllItems%2Easpx%3FRootFolder%3D%252Fkomiteer%252
Flegemiddelkomiteen%252FDokumentbibliotek%2520lager%252FInformasjon%2520om%2520
LIS%252Danbud%252F2018%26FolderCTID%3D0x0120008729410275231245591CBCF04418
E246C%26View%3D%7B4E86623D%2D3B87%2D484B%2DA087%2D07D7BB213411%7D
&DefaultItemOpen=1&DefaultItemOpen=1

Period 01.02.2019–31.01.2021: https://sykehusinnkjop.no/seksjon/avtaler-legemidler/Documents/Hepatitt%20B%20og%20C/Anbefaling%20LIS%201908%20hepatitt%20C.pdf
