# Supplementary material for: The efficacy of integrated hepatitis C virus treatment in relieving fatigue in people who inject drugs: a randomized controlled trial
Source: Subst Abuse Treat Prev Policy. 2023 Apr 24;18:25. doi: 10.1186/s13011-023-00534-1 (PMC10123982; doi:10.1186/s13011-023-00534-1)
Supplement: Supplementary file 2 — Additional file 2. The US-English and the Norwegian versions of the FSS-9. Legends: FSS-9; Nine-items Fatigue Severity Scale. All items in the FSS-9 are ranged as a Likert scale from 1 to 7, where 1 indicates “strongly disagree” and 7 indicates “strongly agree”. [file 13011_2023_534_MOESM2_ESM.pdf]

Additional File 2

I1: My motivation is lower when I am fatigued
I2: Exercise brings on my fatigue
I3: I am easily fatigued
I4: Fatigue interferes with my physical functioning
I5: Fatigue causes frequent problems for me
I6: My fatigue prevents sustained physical functioning
I7: Fatigue interferes with carrying out certain duties and responsibilities
I8: Fatigue is among my three most disabling symptoms
I9: Fatigue interferes with my work, family, or social life

I1: Mitt pågangsmot blir dårligere når jeg er utmattet
I2: Jeg blir fort utmattet ved anstrengelser
I3: Jeg har lett for å bli utmattet
I4: Utmattelse nedsetter min fysiske funksjonsevne
I5: Utmattelse skaper ofte problemer for meg
I6: Utmattelse fører til at jeg har dårlig fysisk utholdenhet over lengre tid
I7: Utmattelse virker negativt inn på mine gjøremål og forpliktelser
I8: Utmattelse er ett av mine tre mest plagsomme symptomer
I9: Utmattelse virker negativt inn på mitt arbeid, min familie og mitt øvrige sosiale liv
