# Supplementary material for: The efficacy of integrated hepatitis C virus treatment in relieving fatigue in people who inject drugs: a randomized controlled trial
Source: Subst Abuse Treat Prev Policy. 2023 Apr 24;18:25. doi: 10.1186/s13011-023-00534-1 (PMC10123982; doi:10.1186/s13011-023-00534-1)
Supplement: Supplementary file 3 — Additional file 3. Aspartate aminotransferase to platelet ratio index. Legends: The figure displays the equation to calculate APRI score. AST upper limit of normal range was defined as 45 IU/Land 35 IU/L. [file 13011_2023_534_MOESM3_ESM.pdf]

Additional File 3

$$APRI = \frac{\frac{AST\ level\ (\frac{IU}{L})}{AST\ (Upper\ limit\ of\ Normal)\ (\frac{IU}{L})}}{Platelet\ Count\ (\frac{10^9}{L})} x\ 100$$
