# Supplementary figures and images for: The efficacy of integrated hepatitis C virus treatment in relieving fatigue in people who inject drugs: a randomized controlled trial
Source: Subst Abuse Treat Prev Policy. 2023 Apr 24;18:25. doi: 10.1186/s13011-023-00534-1 (PMC10123982; doi:10.1186/s13011-023-00534-1)

Additional File 4

Integrated HCV treatment (baseline) (A)

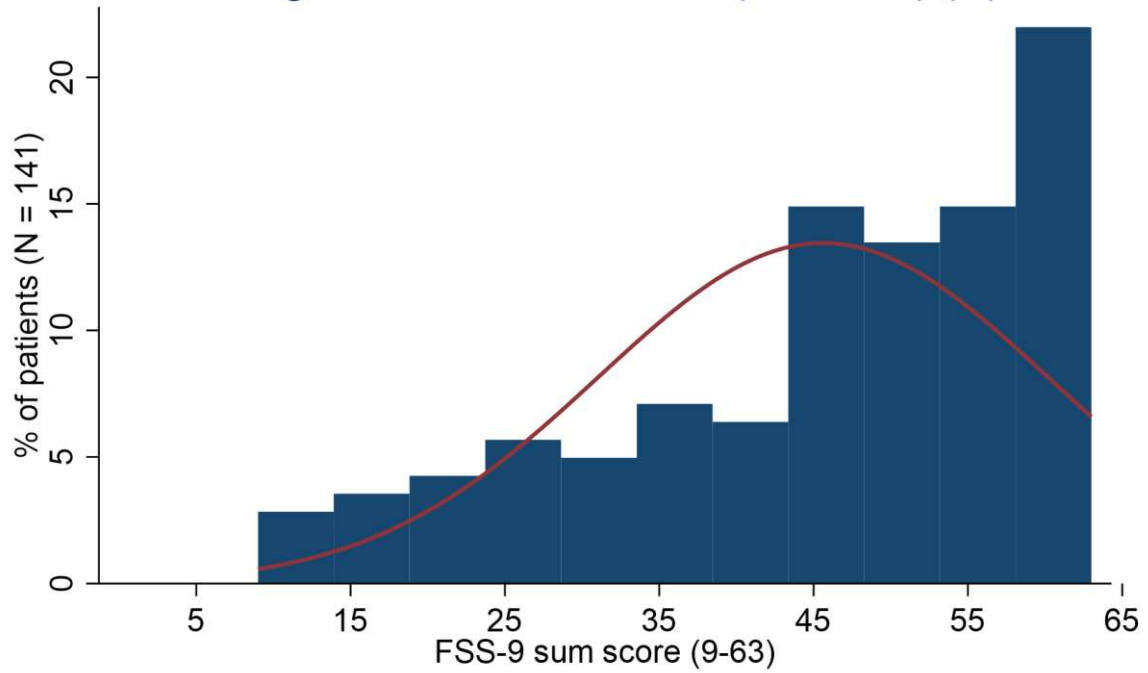

Standard HCV treatment (baseline) (B)

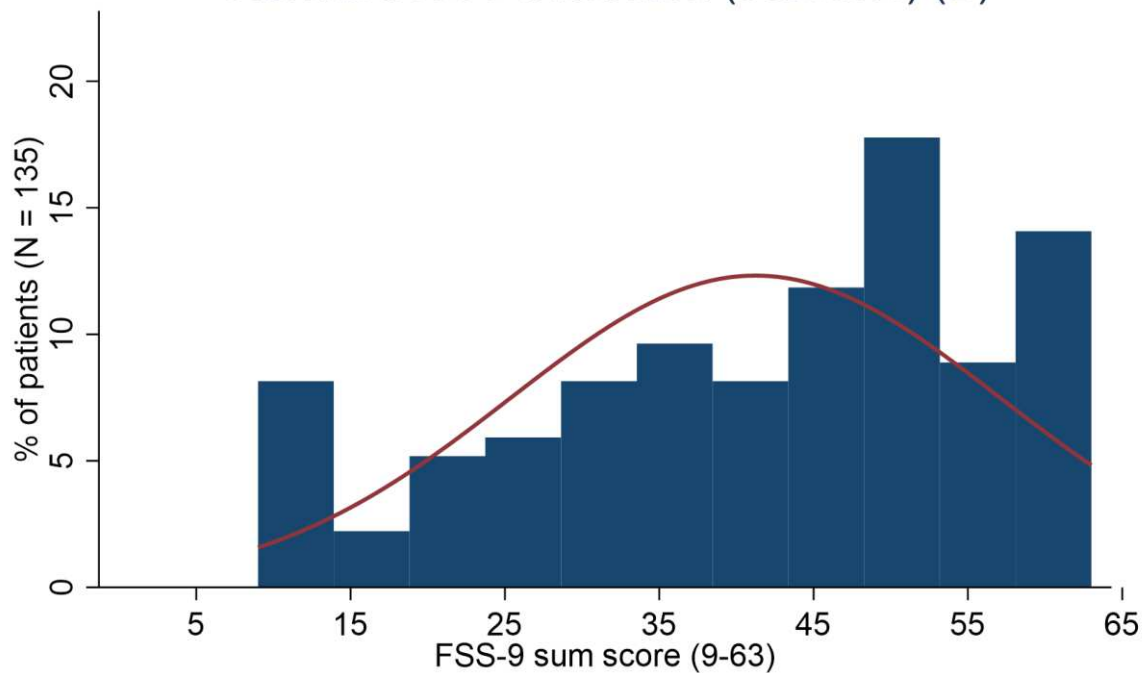

Supplement: Supplementary file 4 — Additional file 4. Distribution of FSS-9 sum scores for integrated HCV treatmentand standard HCV treatmentgroups at baseline. Legends: The two graphsdisplay the FSS-9 sum scores for integrated HCV treatmentand standard HCV treatmentat baseline. The red lines demonstrate the distribution of the FSS-9 sum scores with skewness –0.8and –0.5and kurtoses 2.7 and 2.3. The FSS-9 sum scores ranges from 9 points, no fatigue, to 63 points, worst fatigue. [file 13011_2023_534_MOESM4_ESM.pdf]

Additional File 7

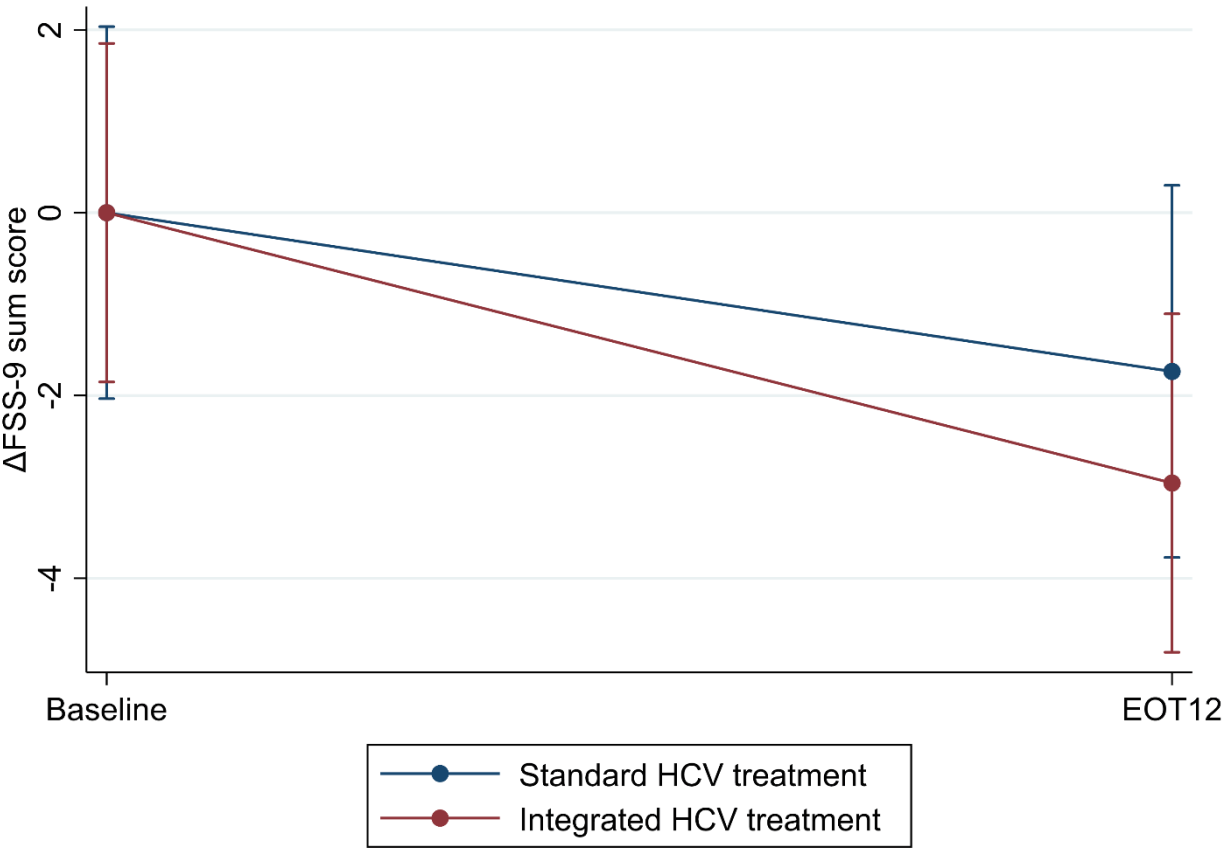

Supplement: Supplementary file 7 — Additional file 7. A linear prediction of changes in FSS-9 sum scores from baseline to EOT12. Legends: The figure displays the linear predictionincluding 95 % confidence intervals of changes in FSS-9 sum scorefrom baseline to EOT12 for integrated and standard HCV treatment groups. EOT12: 12 weeks after the end of HCV treatment; FSS-9: Nine-item fatigue severity scale; HCV: Hepatitis C virus. [file 13011_2023_534_MOESM7_ESM.pdf]

Additional File 8

### Integrated HCV treatment

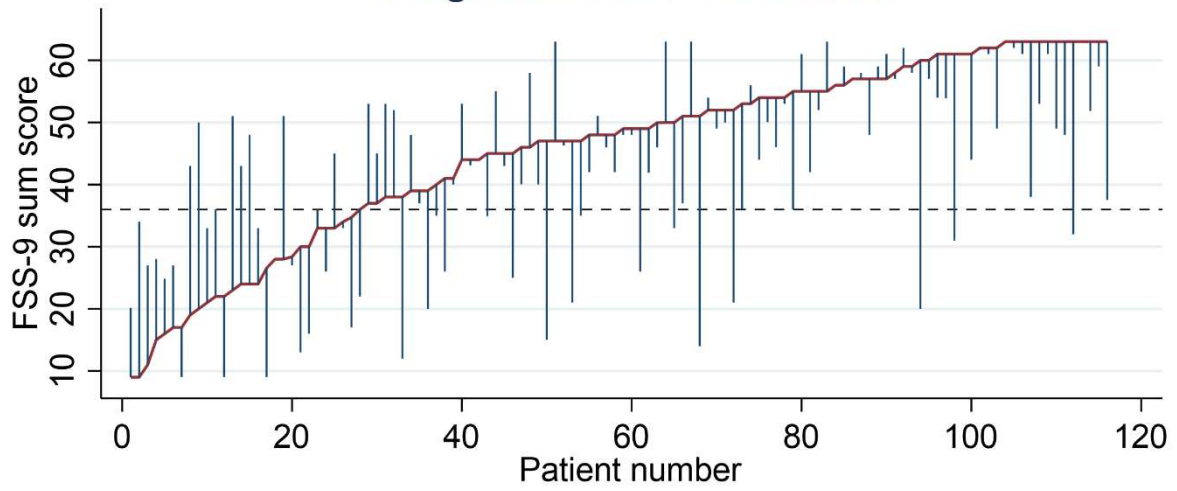

### Standard HCV treatment

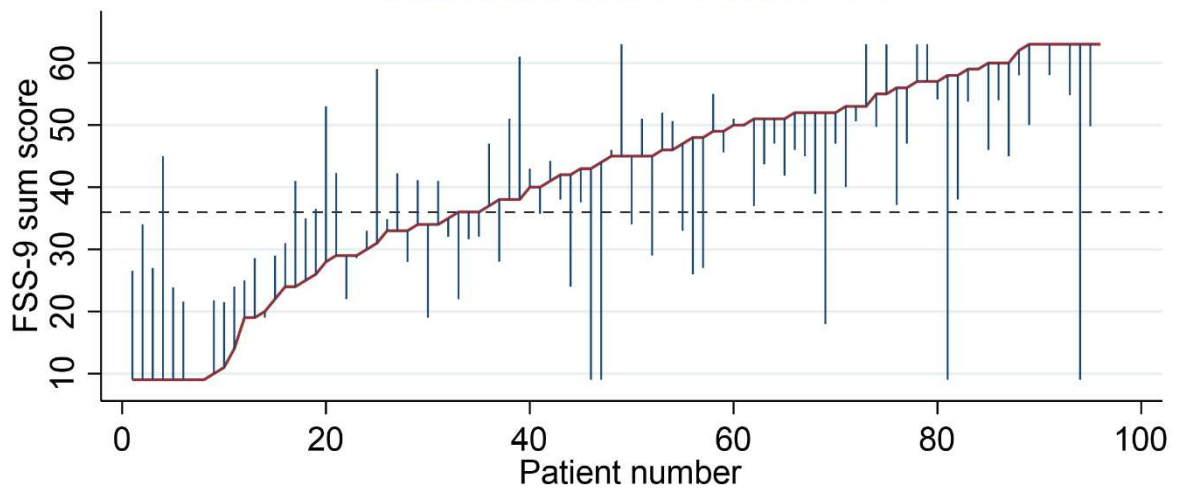

Supplement: Supplementary file 8 — Additional file 8. Pen’s parades of FSS-9 sum scores at baseline and EOT12. Legends: The figures display participants who received integrated and standard HCV treatment and were included in the per-protocol analysis. The graphs demonstrate the FSS-9 sum scores at baseline/prior to the HCV treatment and EOT12. The red line represents the FSS-9 sum scores at baseline when the participants are in sorted order by FSS-9 sum scores to highestscore). The blue spikes demonstrate the FSS-9 sum score at EOT12. The length of the spikes mark the changes in the FSS-9 sum score from baseline to EOT12. Participants without spikes did not complete FSS-9 assessment at EOT12. The dotted line demonstrates the cut-off value for severe fatigue. EOT12: 12 weeks after the end of treatment; FSS-9: Nine-item Fatigue Severity Scale. [file 13011_2023_534_MOESM8_ESM.pdf]
