# Supplementary material for: The efficacy of integrated hepatitis C virus treatment in relieving fatigue in people who inject drugs: a randomized controlled trial
Source: Subst Abuse Treat Prev Policy. 2023 Apr 24;18:25. doi: 10.1186/s13011-023-00534-1 (PMC10123982; doi:10.1186/s13011-023-00534-1)
Supplement: Supplementary file 5 — Additional file 5. Primary end point analyses of mean and sum scores of FSS-9 at baseline and EOT12 Legends: The table displays the mean and sum scores of FSS-9 at baseline and EOT12 among participants who were included in the intention-to-treat and per-protocol analyses, respectively. The FSS-9 sum score ranges from 9 points, no fatigue, to 63 points, worst fatigue. Each item was ranged on a Likert scale from 1 point, no fatigue, to 7 points, worst fatigue. EOT12: 12 weeks after the end of treatment; SD: Standard deviation. [file 13011_2023_534_MOESM5_ESM.pdf]

Additional File 5

	Mean score of all items (SD)	***Sum score of all items (SD)***
Intention-to-treat analyses		
Integrated HCV treatment, baseline (N = 141)	5.1 (1.6)	46 (15)
Standard HCV treatment, baseline (N = 135)	4.6 (1.8)	41 (16)
Integrated HCV treatment, EOT12 (N = 141)	4.7 (1.6)	42 (15)
Standard HCV treatment, EOT12 (N = 135)	4.5 (1.6)	40 (14)
Per-protocol analyses		
Integrated HCV treatment, baseline (N = 116)	5.1 (1.6)	46 (15)
Standard HCV treatment, baseline (N = 96)	4.6 (1.8)	41 (16)
Integrated HCV treatment, EOT12 (N = 116)	4.7 (1.6)	43 (15)
Standard HCV treatment, EOT12 (N = 96)	4.4 (1.7)	39 (15)
