# Supplementary material for: The efficacy of integrated hepatitis C virus treatment in relieving fatigue in people who inject drugs: a randomized controlled trial
Source: Subst Abuse Treat Prev Policy. 2023 Apr 24;18:25. doi: 10.1186/s13011-023-00534-1 (PMC10123982; doi:10.1186/s13011-023-00534-1)
Supplement: Supplementary file 6 — Additional file 6. Linear mixed model of ΔFSS-9 sum score from baseline to EOT12 for integrated HCV treatment (per-protocol) (number of participants = 212, number of observations: 424). Legends: The table displays a linear mixed model analysis (Restricted Maximum Likelihood) regression of the impact of integrated HCV treatment on changes in FSS-9 sum scores (ΔFSS-9 sum scores) from baseline to EOT12 (per-protocol analysis), adjusted for acheiving SVR at EOT12. The FSS-9 sum score ranges from 9 points, no fatigue, to 63 points, worst fatigue. EOT12: 12 weeks after the end of HCV treatment; FSS-9: Nine-item fatigue severity scale. [file 13011_2023_534_MOESM6_ESM.pdf]

Additional File 6

	Effect estimates	
	**Coefficient (95 % CI)**	***p*-value**
Time trend	–0.8 (–5.1;3.4)	0.700
ΔFSS-9 sum score from baseline to EOT12		
Standard HCV treatment	0.0 (ref.)	-
Integrated HCV treatment	–1.0 (–5.0;3.0)	0.633
Achieving SVR	–1.2 (–5.4;3.0)	0.573
