# Supplementary material for: The efficacy of integrated hepatitis C virus treatment in relieving fatigue in people who inject drugs: a randomized controlled trial
Source: Subst Abuse Treat Prev Policy. 2023 Apr 24;18:25. doi: 10.1186/s13011-023-00534-1 (PMC10123982; doi:10.1186/s13011-023-00534-1)
Supplement: Supplementary file 9 — Additional file 9. Linear mixed model of ΔFSS-9 sum scores from baseline to EOT12 for integrated HCV treatment (intention-totreat, sensitivity analysis without computed data), adjusted for SVR at EOT12 (number of participants = 189, number of observations: 378). Legends: The table displays a linear mixed model analysis (Restricted Maximum Likelihood) regression of the impact of integrated HCV treatment and SVR on changes in FSS-9 sum scores (ΔFSS-9 sum score) from baseline to EOT12 (intention-to-treat analysis without computed data by the expectation–maximization algorithm). The FSS-9 sum score ranges from 9 points, no fatigue, to 63 points, worst fatigue. EOT12: 12 weeks after the end of HCV treatment; FSS-9: Nine-item fatigue severity scale; SVR: Sustained virological response. [file 13011_2023_534_MOESM9_ESM.pdf]

Additional File 9

	Effect estimates	
	**Coefficient (95 % CI)**	***p*-value**
Time trend	0.1 (–5.3;5.5)	0.969
ΔFSS-9 sum score from baseline to EOT12		
Standard HCV treatment	0.0 (ref.)	-
Integrated HCV treatment	–2.4 (–7.0;2.3)	0.313
Achieving SVR	–1.6 (–6.5;3.8)	0.603
