# Supplementary material for: The efficacy of integrated hepatitis C virus treatment in relieving fatigue in people who inject drugs: a randomized controlled trial
Source: Subst Abuse Treat Prev Policy. 2023 Apr 24;18:25. doi: 10.1186/s13011-023-00534-1 (PMC10123982; doi:10.1186/s13011-023-00534-1)
Supplement: Supplementary file 10 — Additional file 10. Linear mixed model of ΔFSS-9 sum scores from baseline to EOT12 for integrated HCV treatment, adjusted for SVR . Legends: The table displays a linear mixed model analysis regression of the impact of integrated HCV treatment and SVR on changes in FSS-9 sum scoresfrom baseline to EOT12. The FSS-9 sum score ranges from 9 points, no fatigue, to 63 points, worst fatigue. EOT12: 12 weeks after the end of HCV treatment; FSS-9: Nine-item fatigue severity scale; SVR: Sustained virological response. [file 13011_2023_534_MOESM10_ESM.pdf]

Additional File 10

	Effect estimates	
	**Coefficient (95 % CI)**	***p*-value**
Time trend	3.7 (–3.8;11.2)	0.330
ΔFSS-9 sum score from baseline to EOT12		
Standard HCV treatment	0.0 (ref.)	-
Integrated HCV treatment	0.9 (–4.1;6.0)	0.715
Achieving SVR	–7.4 (–14.6; –0.2)	0.044
