# Supplementary material for: The efficacy of integrated hepatitis C virus treatment in relieving fatigue in people who inject drugs: a randomized controlled trial
Source: Subst Abuse Treat Prev Policy. 2023 Apr 24;18:25. doi: 10.1186/s13011-023-00534-1 (PMC10123982; doi:10.1186/s13011-023-00534-1)
Supplement: Supplementary file 11 — Additional file 11. Linear mixed model of the association between sociodemographic factors, injecting drug use, and drug use and FSS-9 sum score. Legends: The table displays a linear mixed model analysis regression of the impact of sociodemographic factors, injecting drug use, and drug use on FSS-9 sum scores at baseline and from baseline to EOT12in the study sample. The FSS-9 sum score ranges from 9 points, no fatigue, to 63 points, worst fatigue. “Educational attainment” was defined as the highest level of education completed. “Injecting substance use” was defined as having injected any substance at least once during the 12 months leading up to the first health assessment. Drug use was categorized according to the use during the past year. Frequent drug use was defined as consuming at least one of the drugs in the five drug classes more than weekly during the year leading up to the first health assessment. Participants who did not use drugs or used them less than weekly during the year were categorized as having “no frequent use of drugs”. Missing values were identified in 1.4% of FSS-9 scores, 1.1 % of educational attainment, 2.5 % of injecting drug use and 3.3 % of drug use at baseline and 29.9% of FSS-9 score at EOT12, and all were handled as “missing at random” and replaced with estimated values using expectation- maximization algorithm. Except for the “achieving SVR” predictor, we kept all the predictor variables constant at the baseline level in predicting changes in the FSS-9 sum scores from baseline to EOT12. To explore whether predictors predicted changes in the FSS-9 score from baseline to EOT12, the interaction between these factors and timeand EOT12) were added. EOT12: 12 weeks after the end of HCV treatment; FSS-9: Nine-item fatigue severity scale; HCV: Hepatitis C virus; SVR: Sustained virological response. [file 13011_2023_534_MOESM11_ESM.pdf]

Additional File 11

	Baseline		Time trends (from baseline to EOT12)	
	Coefficient (95 % CI)	*p*-value	Coefficient (95 % CI)	*p*-value
Time (from baseline to EOT12)	-	-	5.1 (-4.2;14.4)	0.281
Sex				
Female	4.2 (-5.1;13.5)	0.374	2.0 (-2.1;6.1)	0.340
Age groups				
18-<30	0.0 (ref.)		0.0 (ref.)	
30-<40	1.1 (-5.0;7.2)	0.721	-5.8 (-11.9;0.2)	0.060
40-<50	3.9 (-2.3;10.0)	0.219	-7.4 (-13.5;-1.3)	0.019
50-<60	-1.4 (-7.9;5.0)	0.667	-4.2 (-10.7;2.2)	0.197
≥ 60	5.8 (-4.1;15.8)	0.247	-7.7 (-17.6;2.2)	0.129
Educational attainment				
Not completed primary school	0.0 (ref.)		0.0 (ref.)	
Primary school (9 years)	-3.2 (-10.3;3.8)	0.370	-0.1 (-7.1;6.9)	0.983
High school (12 years)	-7.5 (-14.7;-0.3)	0.040	0.6 (-6.5;7.8)	0.860
≤ 3 years of college or university	-5.9 (-14.7;3.5)	0.227	4.5 (-4.5;13.5)	0.321
> 3 years of college or university	-7.9 (-22.2;6.3)	0.276	-3.5 (-17.8;10.7)	0.626
Injecting drug use	2.3 (-1.6;6.2)	0.240	-3.7 (-7.6;0.2)	0.062
Frequent drug use				
Alcohol	0.5 (-3.6;4.5)	0.889	-1.0 (-5.0;3.0)	0.613
Benzodiazepines	4.1 (0.3;7.9)	0.036	0.8 (-3.0;4.5)	0.687
Cannabis	-0.5 (-4,1;3.1)	0.792	3.5 (-0.1;7.1)	0.059
Opioids	2.7 (-2.9;8.4)	0.338	-1.8 (-7.4;3.7)	0.519
Stimulants (amphetamines and cocaine)	-6.0 (-9.7;-1.5)	0.008	-2.6 (-6.7;1.5)	0.211
Achieved SVR	-	-	-1.1 (-4.7;2.4)	0.523
